# Experts’ opinions on terminology for complementary and integrative medicine – a qualitative study with leading experts

**DOI:** 10.1186/1472-6882-12-218

**Published:** 2012-11-14

**Authors:** Christine Holmberg, Benno Brinkhaus, Claudia Witt

**Affiliations:** 1 Institute for Social Medicine, Epidemiology, and Health Economics, Charité University Medical Center, Luisenstr 57, Berlin, 10117, Germany; 2 University of Maryland School of Medicine, Center for Integrative Medicine, Baltimore, MD, USA

**Keywords:** Integrative medicine, Complementary and alternative medicine, Terminology, Health care delivery system

## Abstract

**Background:**

Integrative medicine (IM) is currently the most commonly used term to describe the integration of complementary and alternative medicine (CAM) into conventional medicine. In the definitions of IM the most important feature is the focus on evidence as crucial factor for therapeutic decision-making. However, there are discussions on the term “integrative medicine” with the most notable critique from within CAM that it describes the integration of complementary methods into conventional institutions and into a “conventional framework of thinking”. The aim of this qualitative study was to understand the thoughts of leading experts on IM and on the scientific debate in the field as well as their personal opinions about terminology in general.

**Methods:**

We have conducted semi-standardized interviews with ten leading experts in the field of CAM and integrative medicine in the USA, England, and Germany, who have had leading positions at medical schools or the NIH in 2010 and 2011. Interviews were recorded, transcribed and analyzed using content analysis with the qualitative analysis software maxqda.

**Results:**

Overall the current terminology was seen as a problem, although most experts agreed that the term “integrative medicine” (IM) described well what they do or they think is useful for medical care. The terminology debate was discussed from four perspectives: 1) from the perspective of medical practice, 2) from the perspective of research, 3) from the perspective of public relations, and 4) from the perspective of health care delivery. These perspectives may be used to evaluate the appropriateness of different terms in use in the field. When interviewees discussed the terminology question, they also discussed the type of health care system they envisioned. Such reflections led the interviewees to caution about too narrow a focus on the terminology question. The question of naming was one about influencing and changing medicine.

**Conclusion:**

The discussion of the experts demonstrated that the discussion about terminology is an important debate about the shaping of medicine. The experts discussed terminology in the light of "how health care systems" should look like in the future.

## Background

Today a shift is taking place in health care debates away from disease orientation towards prevention, wellbeing [1] and patient orientation [2-4]. This reorientation towards patient-centered care led to a recognition of a high prevalence of patients’ use of complementary and alternative medicine (CAM). This fact is increasingly accepted by conventional medical institutions who now offer CAM services across the U.S. and Europe [5-9]. The internationalization and institutionalization of medical traditions outside of conventional medicine led to the introduction of “umbrella terms” such as CAM and integrative health care to capture the range and diversity of methods and philosophies from different traditions and countries [10]. In the early 2000s, CAM had gained widespread recognition in the medical research field [10-12]. At the same time, new terms were introduced that aimed to capture the increasing use of CAM in conventional medicine, particularly the term integrative medicine (IM). The term, however, was not readily adopted in the CAM research field, as CAM was already an umbrella term that had been difficult to establish because it encompasses such a diverse set of practices, traditions, and philosophies depending on regional area and country [13]. Opponents were hesitant to open up a new discussion [11] and were not convinced that new terminology was necessary [14]. The purpose of this paper is to present, in a synthesized manner, an analysis of opinions of some of those who have led the introduction of CAM into academic medicine in order to understand the significance of terminology in the endeavor to integrate CAM into academic medicine and into the health care delivery system.

Overall, the terminology debate revolves around the question of whether or not IM defines a new medical approach that goes beyond CAM [15,16]. Dobos, a physician who introduced IM to Germany, described the basic concept of IM as “the combination of mainstream with Complementary and Alternative Medicine (CAM) supposedly leading to synergistic therapeutic effects” [16], thus, explicitly including CAM within IM. He argues that IM seeks scientific explanations for phenomena found in CAM such as detoxification. IM is therefore built upon scientific evidence, which will eventually change conventional medicine. IM enhances conventional medicine by adding “tools to the tool box”; as well, it changes the focus of care to prevention and self-healing abilities [16]. Such a concept is strongly opposed by others including Walach [9,17], who argues that IM is not the changing of conventional medicine but the taming of CAM [17]. He refutes the claim that a changed medicine will evolve out of the integration of CAM treatments into conventional medicine because the integration is not a conceptual one but one based on a limited understanding of the concept of evidence. If CAM is continued to be understood as “the other” that challenges conventional medicine, then true change becomes possible.

A range of other terms have been named in the literature such as complementary integrative medicine [18]. These are not currently in widespread use. The debate, both within the scientific community and in the public realm, reveals that it is not *just* semantics that are discussed. It is a highly politicized and contentious issue that is centered around a vision and an understanding of what health care delivery should be [19]. It is a timely and important debate within the CAM scientific community due to the growth of CAM as an international research field, which necessitates a clear understanding of what is meant by different terms used [10,20-22]. In light of the understanding that the terminology debate is highly politicized, we have conducted an in-depth interview study with leading IM experts addressing their opinions on the terminology debate.

## Methods

### Design

In 2010 and 2011 we conducted a qualitative interview study with ten experts in the field of CAM and IM [23]. The study had two aims 1) to identify factors for successful integration of CAM into academic medicine and 2) to elicit experts’ opinions on the on-going terminology debate. For the purpose of the present paper, we analyzed the answers to the question in the interview guideline which was targeted at terminology. This specific question was phrased as: “If it comes to terminology, which term is the best from your point of view, CAM, IM, or complementary integrative medicine (CIM)? And how important is the terminology for integration of CAM into conventional medicine?”

### Sample

For the overall study, interview subjects were selected based on their experience with IM or CAM at medical schools for at least 10 years, an international reputation in IM, and visibility on international congresses and in academic associations. All interviewees were head or director of a center/clinic or program. One center provided the opportunity to interview two experts, one with a research background and the other with an additional strong clinical background. In addition, an expert in the field of CAM at the National Institutes of Medicine was included in the study sample.

The purposive sample was used to elicit opinions on terminology because all participants had actively worked towards integrating CAM into conventional medical institutions. However, not all of them were active in introducing new terminology into the research arena even though all of them worked towards the goal of integrating CAM into conventional medicine. The intention of the sample was not to obtain a representative view but to understand the specific view of terminology from those who moved the integration of CAM in medical academic institutions forward.

### Data collection and analysis

All interviews were led by one interviewer trained in interviews for qualitative research (CW) from August 2010 to March 2011. They were then transcribed and uploaded in MAXQDA 10 for further analysis. For the purpose of this paper, a thematic analysis of the terminology question was conducted. Coding took place in several rounds. First, one of the authors (CH) selected all sections of the interview that referred to terminology. Then an inductive approach was used to code the content of these selections (CH). Each segment was coded according to themes present in the material. Codes and coding were discussed within the research team (CH and CW). Finally, core categories were created based on the ensuing analysis. To ensure intersubjectivity and grounding of results, materials, research process, and results were discussed in a qualitative research group.

### Ethics

IRB approval is not required for this type of expert interview study in Berlin. The study was approved by the responsible authority at the Charité University Medical Center (Charité data protection officer). Written informed consent was provided by all of the interview partners.

## Results

The analysis of the materials revealed that the interviewees perceive the terminology debate from different perspectives: 1) from the perspective of medical practice, 2) from the perspective of research, 3) from the perspective of public relations, and finally, 4) from the perspective of health care delivery. If interviewees argued from the perspective of practice or research, they overall favored integrative medicine (IM). If they looked at the terminology debate from a public relations perspective, they perceived complementary and alternative medicine (CAM) as easier to understand for those outside the field. Finally, some were not happy with any of the umbrella terms and were seeking out new terms altogether that encompassed the entire medical health care system.

1)The perspective from medical practice

Most of the interviewees considered IM the best term because it portrayed the integration of the practice of CAM into conventional medical institutions. IM was seen as a descriptor of the physician’s role and the activities of conventional medical institutions. From the perspective of patients’ behavior in medical care, both IM and CAM were seen as adequate descriptors because both demonstrated that many patients used conventional medicine and other therapies in complementary or integrative ways.

a)IM as descriptor of a physician’s role

Some of the interviewed medical doctors discussed the terminology question from the perspective of their work. As medical doctors trained in conventional medicine, they regularly considered conventional treatment options as well as treatment options from other medical traditions for their patients. In the opinion of some of the interviewees, this was precisely what IM stood for.

“It’s what we do. When I do my grand rounds, I first think about the patient’s conventional medication and diagnostics, and then I think about the other, it’s really integrative.”(MD, Germany)

Because of this integration of conventional medicine and other medical traditions in their everyday work, some of the MDs did not see CAM as an appropriate umbrella term. The descriptor “complementary” was perceived to limit the practitioner to the complementary part of therapy and neglect the conventional expertise of MDs who practiced both.

“*What I don’t like about the term complementary medicine is that this in fact often limits one to complementary medicine. And many of us have a good education in conventional medicine.”* (MD, Germany)

b)IM as descriptor of conventional medical institutions

Some interviewees argued from an institutional perspective. Their respective institutions offered conventional medicine and CAM concurrently. Interviewees proclaimed that such offers were important for the institutions because it tied patients to them who may not otherwise come. Such an “integrative” offer provided patients the choices they requested. Thus, from the perspective of conventional medical institutions, integrative medicine seemed the most viable term.

“We do want to offer the full range of choices. But that is (…) happening at medical centers throughout the country, in cancer in particular.”(PhD, USA)

c)CAM and IM as a descriptor of patients’ behaviors

In the views of the interviewees, patients did not choose between CAM therapies and conventional medicine. Normally, they chose from all sorts of medical traditions, including conventional medicine and complementary treatments. Such a behavior was seen to be encompassed by both the term IM and by the term CAM.

Again, IM was seen as the integrated work of CAM and conventional medicine conducted in medical centers. This was seen to reflect patients’ wishes.

*“Integrative medicine. In the way that they’re doing it in the U.S. That’s what patients really want, that’s what all our qualitative data on CAM tell us, this is patient-led, this is a uniquely patient-led process*.” (MD, UK)

The same argument was used to argue for the term CAM as it stressed the complementary aspect of different medical traditions. Again, it was seen as what patients did. They used different medical traditions to treat a condition.

“*Patients don’t go alternative, patients are really going together.*” (PhD, USA)

2)The perspective of research and academia: The vagueness of IM

Interestingly, interviewees portrayed the term IM as a vague term that others outside the CAM field did not understand. This vagueness was seen as an advantage of IM.

*“Yes, absolutely. Because it’s so vague no one knows what it means and we can all define it. For most, it means integrating conventional with complementary medicine. For others, it conveys the concept of treating people as “whole” individuals, integrating mind, body and spirit. But yes, people do really like the term. And sometimes we’ll say complementary and integrative, but we also do want to offer the full range of choices. But that is happening actually at medical centers throughout the country, for cancer in particular.”* (PhD, USA)

Thus, the vagueness meant that it needed to be defined by those shaping the field. Such a term holds the promise to go beyond old debates and worries concerning medical traditions outside conventional medicine, if conventional medicine is seriously part of integrative medicine.

*Integrative medicine is an appropriate term because it does not exclude any serious medical approach. The term is somewhat vague on purpose. (…) Integrative medicine should indicate the combination of conventional and complementary medicine indeed. If someday integrative medicine would be considered a simple synonym for alternative medicine then we need to change it*.” (MD, Germany)

At the same time, the worry was voiced that the vagueness of the term would discredit the field because others may view it as a “Trojan horse” and would develop even bigger resentments against CAM.

“*For the first time it now happened to me that integrative medicine was discussed in a negative way with me. They saw it [IM] as a Trojan horse through which methods that cannot be taken seriously are introduced into medical schools.*” (MD, Germany)

To counter worries in conventional medicine about CAM and IM, one interviewee suggested institutionalising IM through the establishment of a medical specialisation in “IM” for MDs. This would protect the field from being undermined by treatments that have not shown to be safe and effective.

However, such an approach was not seen without problems. There was some concern that the effectiveness of CAM and the dichotomy between CAM and conventional medicine had some benefits that were lost under the label of IM.

“*Experience shows that if one tries to come into dialogue with conventional medicine, one needs to leave the theoretical underpinnings [of CAM] behind (…), because it cannot be evaluated scientifically (…). However, [the theoretical background] is extremely important. Time and again I have experienced that if you are trying to use only a technique from CAM such as acupuncture it is not as effective (….). I think integrative medicine is good, but we need to find a way to teach integrative medicine and to make it less prone to criticism and I am worried that that will in fact lead to a significant reduction of what CAM has to offer.”* (MD, Germany)

The concern was echoed by another interviewee who suggested that the fight between complementary and conventional medicine may be important to improve and bring research forward.

*“The question is if integrative medicine also has disadvantages. If the polarisation, the discussion, and maybe the disruption, may be crucial for scientific development.”* (MD, Germany)

3)From the perspective of public relations: CAM

Many of the interviewees agreed that IM was a vague term that was not understood by lay people. This was also an argument against the term IM. If one wanted others to understand what one was doing, the term CAM might provide more clarity.

“*because people then know what we are talking about. If we wrote, this is a masters in integrative medicine nobody knows what you are saying. So we are using complementary and alternative (…), so people know we are talking about the CAM disciplines*.” (PhD, USA)

4)From the perspective of health care delivery

When interviewees discussed the terminology question, they also discussed the type of health care system they envisioned. Such reflections led the interviewees to caution about too narrow a focus on the terminology question. The question of naming was one about influencing and changing medicine. Similarly, interviewees pointed towards a re-orientation of medical care towards healing or patient-centered care.

*“I think they’re [the suggested names] all place holders. You know, until you just can get to where … we have influenced enough that medicine sort of gets back to its, some of its holistic roots, and its healing roots.”* (MD, US)

*“What we need to deliver at the clinic, is what I think we should focus on. Because I think, we can agree on that, and we got real consensus with the patient. I don’t think it matters what you call it. (…) We know what we want to deliver, and we haven’t got a really good way of describing it. But maybe! it’s patient-centred medicine, maybe! we should stop worrying about the complementary medicine, and we should just start talking about our medicine being patient-centred rather than process-driven”* (MD, UK)

In line with the cautioning about fixating on terminology, the problem of restricting oneself by a name was also mentioned. This seemed particularly pertinent because disciplinary boundaries change with time and what may currently be perceived as complementary medicine could later become conventional medicine.

I wouldn’t give it, I would, I, I wouldn’t give it a very descriptive name, (…) But complementary medicine isn’t mentioned. (…)I wouldn’t want to be labeled in a particular way, because who knows, how! we’re going to deal with mindfulness in ten years’ time.” (MD, UK)

## Discussion

The question of how to name a field involves many perspectives that the interviewees considered: the actual practice of medical care, the necessity for others to understand what is meant by a term, and the consideration of the potential effects of a term once chosen. Thus, interviewees consider the naming issue in the context of its usage. At the same time, the perspectives that influenced how they thought about a particular term show that naming is not simply labeling something that exists, it shapes the “something” that is to be named. In this sense, a discussion on terminology often becomes a politicized debate on the shaping of a (medical-research) field.

The importance of the terminology debate and its influence on shaping a field is crucial, especially due to the increasing resentments and campaigns against the trend of including CAM into conventional medical centers [12,24]. Projects are under way that seek to clarify the meaning of terms that are used in the context of the integration of different medical traditions into conventional health care delivery [13]. These projects demonstrate that it is necessary to evaluate the use of terminology within a context and that perhaps umbrella terms such as CAM and IM should not be used in extenso in local contexts. However, these terms are important for a common understanding in research and political discussions [10]. It is precisely in those arenas that medical fields are shaped and developed.

Certainly, the discussion of the experts demonstrated that their intention is to close ideological gaps that exist within health care delivery systems. While some argued that the name is only a “place holder”, it became obvious that this place holder needs to be carefully considered. Since the terminology debate does aim at changing the focus of medical care. For some of the interviewees the vagueness and newness of the term “IM” was seen as a possibility to transcend the dichotomization of ideological debates between conventional medicine and other medical traditions, thus, moving towards a medical system that based its treatment decisions on evidence and not on medical tradition. This in many ways aligns with a general shift in medical practice in which efforts are increasing to ensure that treatments are evidence-based regardless of medical tradition [24,25]. In this regard, IM reflects the ideology and recent movement of evidence-based medicine.

However, the experts moved beyond the focus on evidence in their discussions on terminology and focused on how what health care systems should look like in the future. These visions did include a focus on evidence. Consequently, it went beyond this by introducing concepts such as holism and patient-orientation. One could speculate, based on the analysis of the thoughts the experts voiced on terminology, that IM may be a necessary step towards transcending historical dichotomies. However, eventually IM may be replaced by a health care delivery system that integrates different medical traditions, including different theoretical traditions as understood by Walach.

Interestingly, some of the interviewees suggested CAM as the name that fits best because people understand the term. This is an intriguing argument when we consider that in the early 2000s there was some discomfort with the term “CAM” as it was seen as a hybrid, scientific term that was not understood by lay people [26]. The question that remains open is which of the available terms may best foster the inclusion of CAM in the current health care system. Based on the findings of this and other studies [10], it may well be that the decision on the use of terminology cannot be definite and needs to be contextual. The four perspectives that were presented in this study may provide a framework for the evaluation of suitable terms. However, this needs to be tested further.

Limitations of the study include a highly selective and small sample of IM specialists who come from only three countries. Clearly, CAM and IM are important across many more countries. Similarly, we have chosen a qualitative, in-depth approach with only ten people. A larger sample may have brought to light other aspects on the terminology debate. The sample included not only MDs but also PhDs. It is possible that the terminology debate was more important for MDs who may have to battle ideological concerns in daily patient care more often than PhDs. Inclusion of practitioners and researchers broadened the debate beyond the medical practice to include research and the advantages and disadvantages IM had for science. Other studies suggest that umbrella terms such as IM and CAM are most salient for the research arena [10]. The purpose of this paper was to learn the opinions of those who worked towards establishing CAM in medical schools and funding agencies to understand what is at stake in the terminology debate. In that manner, we were able to show the different perspectives and complexities under which experts discuss terminology. This demonstrated the political nature of the terminology debate. In light of these findings, new studies should be developed to analyze which of the discussed terms may indeed be best to establish the experts’ goals. Such a question should also be addressed from the point of view of those practicing CAM in conventional medicine and should include conventional medical practitioner as well.

This is the first study in which experts who worked towards integrating CAM into conventional medicine were asked about their opinion on the on-going terminology debate in order to understand what perspectives are indeed important to consider in the debate. As we chose to synthesize the results, we were able to demonstrate that the debate is highly politicized and aims towards confronting the strong opposition to CAM from a large segment of the established medical community. We were also able to demonstrate that the experts were aware of the costs such an approach may have.

## Conclusion

Rather than viewing IM as a dogmatic field focused on narrow concepts of evidence, it could be argued that it is a transitional term that can aid in removing barriers and opening up medical practice and research towards new visionary health care delivery. Part of this vision is a clear focus on evidence-building and patient-orientation. Such concepts are at the heart of current developments in medicine and health care delivery that is concerned with changing conventional medical practice. Thus, IM may be the beginning of a general change from conventional medicine towards a true integration of different medical styles and practices including an improvement of the patient-practitioner relationship into the best care patients can receive. Therefore, the terminology debate is about the shaping and development of medical care.

## Competing interests

None of the authors has any conflict of interests with regards to the content of this paper.

## Authors’ contributions

CH has conducted the analysis and interpretation of the data; she has written and revised the manuscript. BB has critically revised the manuscript and given important intellectual content. CW has designed the study, has collected the data, analysed and interpreted the data. Finally, she has critically revised the manuscript and given important intellectual content. All authors read and approved the final manuscript.

## Pre-publication history

The pre-publication history for this paper can be accessed here:

http://www.biomedcentral.com/1472-6882/12/218/prepub
